# Aortic and Mitral Valve Endocarditis—Simply Left-Sided Endocarditis or Different Entities Requiring Individual Consideration?—Insights from the CAMPAIGN Database

**DOI:** 10.3390/jcm13195841

**Published:** 2024-09-30

**Authors:** Carolyn Weber, Mateo Marin-Cuartas, Sems-Malte Tugtekin, Mahmoud Diab, Shekhar Saha, Payam Akhyari, Ahmed Elderia, Florian Muench, Asen Petrov, Hug Aubin, Martin Misfeld, Artur Lichtenberg, Christian Hagl, Torsten Doenst, Klaus Matschke, Michael A. Borger, Thorsten Wahlers, Maximilian Luehr

**Affiliations:** 1 Department of Cardiothoracic Surgery, University of Cologne, 50937 Cologne, Germany; 2 University Department of Cardiac Surgery, Leipzig Heart Center, 04289 Leipzig, Germany; 3 Department of Cardiac Surgery, Heart Center Dresden, 01307 Dresden, Germany; 4 Department of Cardiothoracic Surgery, Friedrich Schiller University Jena, 07747 Jena, Germany; 5 Department of Cardiac Surgery, Herz-Kreislauf-Zentrum (HKZ) Klinikum Herfeld-Rotenburg, 36199 Rotenburg an der Fulda, Germany; 6 Department of Cardiac Surgery, Ludwig Maximilian University Munich, 81377 Munich, Germany; 7 German Centre for Cardiovascular Research (DZHK), Partner Site Munich Heart Alliance, 81377 Munich, Germany; 8 Department of Cardiovascular Surgery, Heinrich-Heine University Duesseldorf, 40225 Dusseldorf, Germany; 9 Department of Thoracic and Cardiovascular Surgery, University of Essen, 45141 Essen, Germany; 10 Department of Cardiothoracic Surgery, Royal Prince Alfred Hospital, Sydney, NSW 2050, Australia; 11 Institute of Academic Surgery, RPAH, Sydney, NSW 2050, Australia; 12 The Baird Institute of Applied Heart and Lung Surgical Research, Sydney, NSW 2050, Australia; 13 Sydney Medical School, University of Sydney, Sydney, NSW 2050, Australia

**Keywords:** infective endocarditis, IE, aortic valve, mitral valve, left-sided IE, cardiac surgery, valve surgery

## Abstract

**Background**: Aortic valve infective endocarditis (AV-IE) and mitral valve infective endocarditis (MV-IE) are often grouped together as one entity: left-sided endocarditis. However, there are significant differences between the valves in terms of anatomy, physiology, pressure, and calcification tendency. This study aimed to compare AV-IE and MV-IE in terms of patient characteristics, pathogen profiles, postoperative outcomes, and predictors of mortality. **Methods**: We retrospectively analyzed data from 3899 patients operated on for isolated AV-IE or MV-IE in six German cardiac surgery centers between 1994 and 2018. Univariable and multivariable analyses were performed to analyze the risk factors for 30 day and 1 year mortality. A Log-rank test was used to test for differences in long-term mortality. **Results**: Patients with MV-IE were more likely to be female (41.1% vs. 20.3%.; *p* < 0.001). Vegetation was detected more frequently in the MV-IE group (66.6% vs. 57.1%; *p* < 0.001). Accordingly, the rates of cerebral embolic events (25.4% vs. 17.7%; *p* < 0.001) and stroke (28.2% vs. 19.3%; *p* < 0.001) were higher in the MV-IE group. Staphylococci had a higher prevalence in the MV-IE group (50.2% vs. 36.4%; *p* < 0.001). Patients with MV-IE had comparable 30 day mortality (16.7% vs. 14.6%; *p* = 0.095) but significantly higher 1 year mortality (35.3% vs. 29.0%; *p* < 0.001) than those with AV-IE. Kaplan–Meier survival analysis showed significantly lower long-term survival in patients with MV-IE (log-rank *p* < 0.001). **Conclusions**: Due to the relevant differences between MV-IE and AV-IE, it might be useful to provide individualized, valve-specific guideline recommendations rather than general recommendations for left-sided IE.

## 1. Introduction

Infective endocarditis (IE) is traditionally often differentiated into right-sided or left-sided IE based on the site of infection. As a result, IE of the aortic and mitral valves is often grouped together as one entity: left-sided endocarditis. However, there are relevant differences between the valves in terms of anatomy, physiology, pressure, and the tendency to calcify. In high-income countries, the most common pathology of the aortic valve is aortic stenosis caused by calcification, while mitral regurgitation is the most common mitral valve pathology caused by leaflet prolapse [1,2].

It is, therefore, not surprising that pathogens such as *Staphylococcus aureus* (*S. aureus*) and enterococci, which have different adherence strategies to host tissues, appear to have different propensities to attach to and colonize different valves [3,4,5,6]. Furthermore, it is known that aortic valve endocarditis (AV-IE) is associated with a higher rate of invasion and occurs more frequently perivalvular, whereas mitral valve endocarditis (MV-IE) is associated with a higher rate of cerebral embolism [1,7].

To better understand the determinants of outcomes and gain a more detailed understanding of IE in the aortic valve compared with the mitral valve, the hypotheses were as follows:

1. Surgical treatment of MV-IE is associated with a higher mortality rate than that of AV-IE.
2. MV-IE itself is an independent risk factor for mortality.

Thus, AV-IE and MV-IE were compared in terms of patient characteristics, pathogen profiles, postoperative outcomes, and predictors of mortality in surgical patients to provide insights into individualized treatment approaches. As the current guidelines only provide recommendations for left-sided endocarditis, the aim was to demonstrate how much these two IE entities differ not only in preoperative profile but also in postoperative outcomes. This could provide a basis for making separate treatment recommendations for these two entities in the future.

## 2. Materials and Methods

### 2.1. Study Design

**Figure 1:**
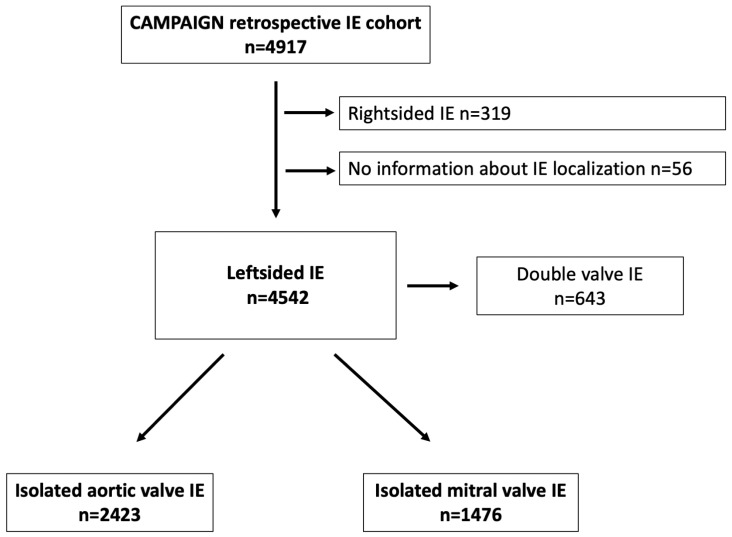
Flowchart of patients with left-sided infective endocarditis and their grouping based on aortic and mitral valve endocarditis. CAMPAIGN, Clinical Multicenter Project for Analysis of Infective Endocarditis in Germany; IE, infective endocarditis.

IE patients whose data entered the “Clinical, Multicenter Project for Analysis of Infective Endocarditis in Germany” (CAMPAIGN) registry underwent surgery in 6 cardiac surgery centers in Germany between 1994 and 2018. There were no formal exclusion criteria. After excluding patients with double valve endocarditis, data from patients with left-sided endocarditis were included in the analysis (Figure 1).

Data collected included demographic data and comorbidities, manifestation of IE according to the recently modified Duke criteria [8] (echocardiographic and microbiological findings), operative data (cardiopulmonary bypass time, ischemia time, concomitant procedures), and relevant postoperative outcomes during inpatient stay. Patients were treated according to the currently valid endocarditis guidelines. For the follow-up, we first examined the medical records to see whether they had been hospitalized again in the meantime or had a recurrence. In addition, all patients and attending physicians were interviewed by telephone.

The follow-up time comprised the time interval between the date of surgery and the date of death or the date of the last contact with the patient (or the corresponding information about the patient’s condition by the attending physician).

### 2.2. Statistical Analysis

Unless otherwise indicated, continuous variables are expressed as mean (+/− standard deviation) or median (interquartile range) according to the normal distribution. The *t*-test was used to compare continuous variables if a normal distribution was present. If the requirements for a normal distribution were not met, the Mann-Whitney-U-test was used. The chi-square or Fisher exact test was used to compare categorical variables on the basis of pre-specified criteria. The chi-square test was used to test two categorical variables when the expected frequencies were greater than 5, and the Fisher exact test was used for expected frequencies of less than 5. Discrete variables are expressed as an absolute number (percentage). Missing data were not imputed and were assumed to be missing at random. The log-rank test was used to test for differences in long-term mortality between MV-IE and AV-IE. The resulting *p*-values and additional Kaplan–Meier survival curves are presented. Inverse Kaplan–Meier was used to calculate the median survival. Potential risk factors for 30 day mortality and 1 year mortality were assessed using logistic regression or Cox regression, respectively. After univariable analysis, all variables with a *p* value of less than 0.1 were included in the multivariable model using forward selection (likelihood ratio, pin = 0.05). The results are reported as odds ratios for 30 day mortality or hazard ratios for 1 year mortality with corresponding 95% confidence intervals and *p*-values. To reduce potential treatment selection bias in comparing the outcomes between groups, inverse probability weighting (IPW) was performed. Propensity scores were calculated through logistic regression analysis using the following variables: age, sex, BMI, hypertension, diabetes, hyperlipidemia, smoking, COPD, peripheral artery disease, pulmonary hypertension, preoperative CKD, preoperative hemodialysis, preoperative stroke, coronary artery disease, myocardial infarction, septic cerebral embolism, LVEF, alcohol abuse, preoperative ventilation, and Staph. aureus, streptococci, enterococci, vegetation, and PVE. Inverse propensity scores were created, and weighting was performed for all patients. To estimate causal effects, a generalized estimation equation was used. All *p*-values reported are 2-sided. Statistical analyses were performed using SPSS Statistics version 28 (Armonk, NY, USA: IBM Corp).

### 2.3. Ethical Statement

The study protocol was approved by institutional ethics committees (Ethics Committee of the Medical Faculty, University of Cologne, 17-407).

## 3. Results

This multicenter retrospective analysis included 3899 consecutive patients with isolated aortic or mitral valve IE. As shown in the flow diagram (Figure 1), from the retrospective CAMPAIGN cohort (4917 IE patients), data from 4542 patients with left-sided IE were analyzed. After excluding 643 patients with concurrent aortic and mitral valve endocarditis, data from 2423 patients with AV-IE and 1476 patients with MV-IE were included in further analyses.

### 3.1. Characteristics of Patients with MV-IE versus AV-IE

**Figure 2:**
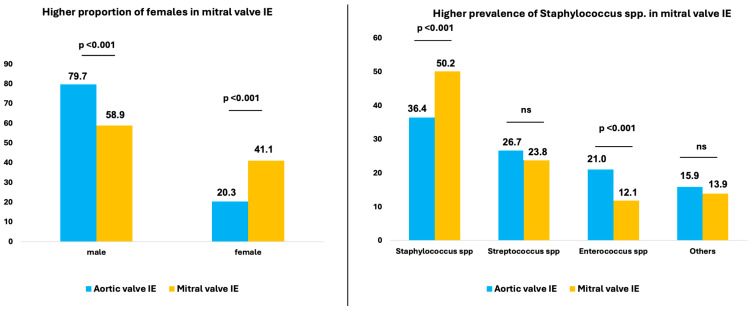
Distribution of sex and causative microorganisms in patients with aortic and mitral valve IE. IE, infective endocarditis.

Patients with MV-IE were slightly older at 66.0 [56.0–74.0] compared to 65.0 [53.0–73.0] years (*p* = 0.013). Overall, male patients were more likely to have IE, but the proportion of female patients with MV-IE was twice that of AV-IE (41.1% vs. 20.3%.; *p* < 0.001). In terms of comorbidities, patients with MV-IE were more likely to have diabetes (29.1% vs. 24.5%; *p* = 0.001) and pulmonary hypertension (21.3% vs. 14.5%; *p* < 0.001) and were more likely to be ventilated preoperatively (11.3% vs. 7.5%; *p* < 0.001) (Table 1 and Figure 2).

Vegetation was detected more frequently in patients with MV-IE than in those with AV-IE (66.6% vs. 57.1%; *p* < 0.001). Accordingly, the rate of cerebral embolic events was higher in patients with MV-IE (25.4%) compared to those with AV-IE (17.7%; *p* < 0.001). Consistent with the higher rates of vegetation and cerebral embolic events, there was an increased prevalence of preoperative stroke in the group of patients with MV-IE (28.2% vs. 19.3%; *p* < 0.001). In contrast, prosthetic endocarditis occurred more frequently in the AV-IE group (16.6% vs. 33.4%; *p* < 0.001) (Table 1).

Staphylococci were the most common causative agents of IE and had a higher prevalence in MV-IE. Staphylococci were found to be the microbial agents in half of the cases with MV-IE (50.2% vs. 36.4%; *p* < 0.001). Enterococci, on the other hand, were significantly more common in AV-IE (12.1% vs. 21.0%; *p* < 0.001) (Table 1 and Figure 2).

### 3.2. Postoperative Outcomes after Surgery for MV-IE versus AV-IE

The postoperative outcomes are shown in Table 2: the postoperative course of patients with MV-IE was more frequently characterized by complications than in AV patients. Thus, patients with MV-IE more often had postoperative stroke (17.7% vs. 14.4%; *p* = 0.011), required hemodialysis (19.3% vs. 13.9%; *p* < 0.001), and were tracheotomized (10.2% vs. 7.0%; *p* < 0.001).

In terms of mortality, patients operated on for MV-IE had comparable 30 day mortality (16.7% vs. 14.6%; *p* = 0.095) compared to patients with AV-IE, but significantly higher 1 year mortality (35.3% vs. 29.0%; *p* < 0.001).

**Figure 3:**
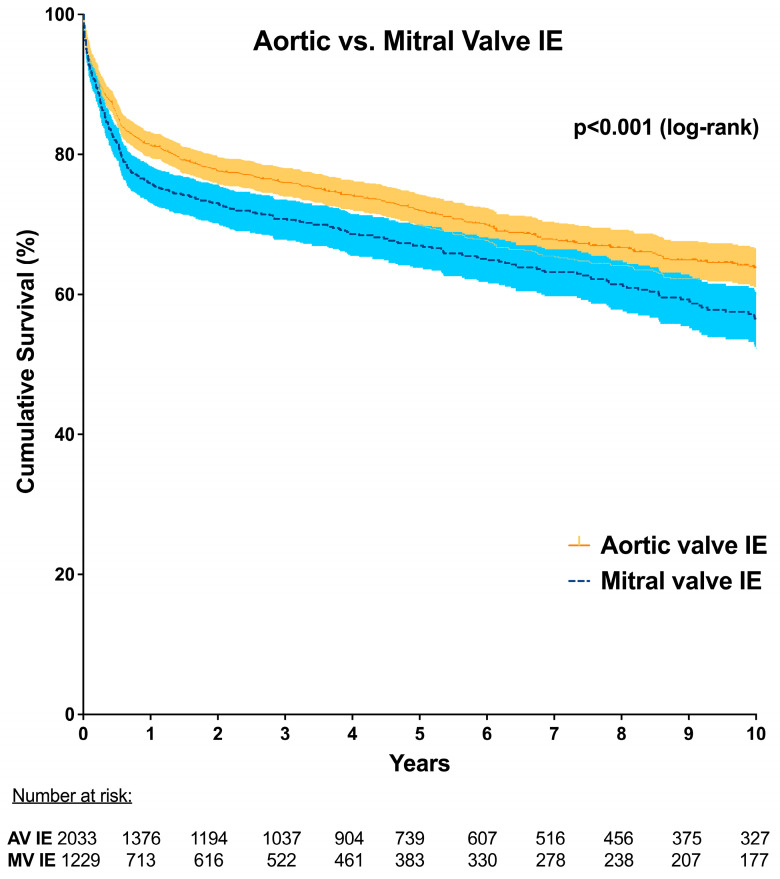
Kaplan−Meier curve showing long-term survival of patients with mitral valve IE compared to aortic valve IE. IE, infective endocarditis.

Kaplan−Meier survival analysis also showed significantly lower long-term survival in patients operated on for MV-IE compared to those operated on for AV-IE (*p* < 0.001) (Figure 3). Follow-up was obtained with a completeness of 86.3% and a median follow-up time of 4.8 [4.5–5.0] years.

### 3.3. Independent Predictors of Mortality for Patients with Left-Sided IE

First, we used multivariable analysis to examine predictors of 30 day and 1 year mortality for the total left-sided endocarditis group (Table 3). Here, we have shown that MV-IE itself was an independent risk factor for 1 year mortality (HR 1.329, 95% CI 1.043–1.693; *p* = 0.021).

We then examined the predictors of 30 day and 1 year mortality separately for AV-IE and MV-IE: this multivariable analysis showed that the predictors of 30 day mortality differed significantly depending on the valve operated on (AV-IE vs. MV-IE) (Table 4).

Staphylococci were the only independent predictor of 30 day mortality in patients with AV-IE, but not in patients with MV-IE. There were also different risk factors for 1 year mortality in the AV-IE and MV-IE groups. For 1 year mortality, staphylococci were independent predictors in the MV-IE group (Table 5).

Even after the possible differences between the groups were reduced when comparing the results using IPW, primary MV-IE was found to be an independent risk factor for 1 year mortality (OR 1.410, CI [1.107–1.795]; *p* = 0.005). For 30 day mortality, MV-IE was not an independent risk factor after IPW.

## 4. Discussion

Our data suggest that patient characteristics, pathogen profiles, and postoperative outcomes differ significantly between AV-IE and MV-IE: patients with MV-IE (i) were significantly younger and more often female, (ii) more often had Staphylococcus-mediated IE associated with more vegetation and higher rates of embolism and stroke, and (iii) had a higher 1 year mortality rate. (iv) MV-IE itself appears to be an independent risk factor for 1 year mortality.

### 4.1. Gender Distribution in MV-IE versus AV-IE

Various studies have already shown that, in general, men are more frequently affected by IE [1,9,10,11,12]. We were able to show that women were significantly more often affected by MV-IE compared to men, which is in line with previous reports [11,12]. Similar to our data, Van Vlasselaer et al. reported a higher proportion of female patients with MV-IE compared with AV-IE (39% vs. 26%; *p* < 0.001) [1]. A study examining sex differences in IE patients showed that female patients were more likely to have mitral valve involvement and more IE-related neurological deficits [9].

For mitral valve surgery, Song and colleagues [13] have demonstrated higher in-hospital mortality for female patients than for male patients. In contrast, Mokhles et al. showed comparable mortality in female and male patients who underwent mitral valve surgery [14].

### 4.2. Risk of Cerebral Embolism in MV-IE Compared with AV-IE

Left-sided IE is associated with a high incidence of embolic events, ranging between 14% and 49% [7,15,16]. Embolic events are among the most common and life-threatening complications that can lead to stroke and occur in 20–40% of IE cases [15,17]. Several factors are associated with increased embolic risk, including vegetation size and mobility, increasing or decreasing vegetation size under antibiotic therapy, certain microorganisms (especially *S. aureus*), previous emboli, and vegetation location at the mitral valve [18,19,20]. In line with this, several studies have identified MV-IE as an independent risk factor for the development of cerebral embolism [7,21,22]. Our data also show that in patients with mitral valve endocarditis, staphylococci are significantly more frequently detectable as microbial agents, which in turn is associated with larger vegetation and more septic emboli. As a result, patients have a longer ICU stay and a poorer 30 day mortality. These are, therefore, relevant complications compared to AV-IE, which should be given appropriate attention.

Furthermore, staphylococcal endocarditis has been shown to be a risk factor for embolization, which is of crucial relevance as the incidence of *S. aureus* IE is increasing [19,23]. Paul et al. found more septic emboli in staphylococci compared with streptococci [24], and *S. aureus* as a causative microorganism for IE could be linked to an increased risk of stroke [16].

In a recent study, Trifunovic et al. investigated the relationship between the microbial agent and the specific cardiac lesions. They were able to show that large vegetation was mostly caused by *S. aureus*, whereas abscesses were more common in Coagulase-negative staphylococci (CoNS) [18]. *S. aureus* has a high propensity to attach to extracellular matrix proteins, fibrin, and platelets, as numerous cell wall-associated factors promote vegetation formation and growth [5,25]. Furthermore, virulent organisms such as *S. aureus* are known to have high adherence and infect valves with minor underlying lesions or even normal valves [26,27]. This might explain why *S. aureus* is particularly found in rarely degenerated mitral valves.

Our data were able to show clear differences between AV-IE and MV-IE regarding microbiological etiology: Staphylococci were the most frequent microbial agents and had a higher prevalence in MV-IE. Enterococci, on the other hand, were found almost twice as frequently as microbial agents in AV-IE compared to MV-IE. Comparable results were reported by Van Vlasselaer et al. in their nationwide registry study that compared native AV-IE with MV-IE [1]. This difference in localization preference between enterococci and *S. aureus* can be explained by the unique flow, pressure, and calcification conditions of the two valves.

### 4.3. Mortality in MV-IE versus AV-IE

Our data show comparable 30 day mortality in MV-IE compared with AV-IE (16.7% vs. 14.6%; *p* = 0.095), but significantly higher mortality up to one year for patients with MV-IE at 35.3% compared with 29.0% in AV-IE (*p* < 0.001). Van Vlasselaer, on the other hand, has reported higher in-hospital mortality for MV-IE than for AV-IE [1]. Ostergaard et al. have also shown that in-hospital mortality was 8.1% for patients with isolated AV-IE, 14.3% for isolated MV-IE, and 18.0% for combined aortic and mitral valve IE (*p* < 0.001) [28]. The odds ratio for in-hospital mortality was higher for patients who underwent cardiac surgery for isolated MV-IE compared with AV-IE [28]. This has also been confirmed by the data of Hussain et al., who found higher mortality for MV-IE compared to AV-IE, and even higher mortality for combined aortic and mitral valve IE [29]. The higher mortality for surgery due to IE in the mitral valve compared to the aortic valve may be due to the fact that radical debridement is often much more difficult and challenging than in the aortic valve due to its localization in the atrioventricular groove. However, our multivariable analysis has shown that MV-IE itself was an independent predictor of mortality up to one year (HR 1.329, 95% CI 1.043–1.693; *p* = 0.021).

### 4.4. Limitations

Our study has several limitations that need to be considered when interpreting the results. Due to its retrospective nature and the associated biases, this study is limited. Due to the multicenter origin of the data and the long study period, there is a certain degree of data heterogeneity between the participating centers. As all institutions were tertiary care centers, our cohort might be influenced by referral bias; therefore, the results of the present study might not be generalizable. To reduce the potential treatment selection bias in comparing the outcomes between groups, inverse probability weighting (IPW) was performed. Furthermore, additional relevant variables that would have allowed a more detailed analysis of the subsequent calculation of endocarditis scores were missing, such as more detailed microbiological information, pre-existing valve disease, information on antibiotic treatment and the time course from diagnosis to surgical treatment, and information on GFR and sPAP. As the causes of death were not recorded in all centers during the follow-up in this retrospective setting, we are unfortunately unable to distinguish whether the patients died from reinfection, surgical complications, or general causes of death. As this was a multicenter retrospective study, there was no uniform protocol for the follow-up in the various centers, so the median follow-up was 4.8 years. Due to the retrospective design and the multicenter approach, there is a lack of data in both groups. Since missing data occur comparatively frequently in both groups, we assume that the data are missing at random and have no relevant influence on the analysis.

Nonetheless, in one of the largest surgical endocarditis collectives, we are able to demonstrate the relevant differences in patients with aortic versus mitral valve IE. We hope that our data will serve as support to provide separate individualized recommendations for patients with aortic and mitral valve endocarditis in the future.

## 5. Conclusions

Patients operated on for MV-IE differed significantly from those operated on for AV-IE in terms of patient characteristics, pathogen profiles, and postoperative outcomes: Patients with MV-IE were more often female and presented with more vegetation, which consequently led to a higher rate of cerebral septic emboli and stroke. In addition, patients with MV-IE had more staphylococcus infections and significantly worse long-term outcomes. MV-IE itself represents an independent predictor of 1 year mortality.

The differences in the preoperative risk profile, particularly in the presence of septic emboli and the increased incidence of staphylococcus infections, indicate that instead of general treatment recommendations for left-sided IE, valve-specific guideline recommendations may be useful in the future.

## Figures and Tables

**Table 1:** Characteristics of patients undergoing surgery for aortic valve IE compared to mitral valve IE.

	Aortic Valve IE (*n* = 2423)	Mitral Valve IE (*n* = 1476)	*p* Value
Age (years)	65.0 [53.0–73.0]	66.0 [56.0–74.0]	**0.013** #
Sex (%)			
male	79.7% (1930/2423)	58.9% (869/1476)	**<0.001** †
female	20.3% (493/2423)	41.1% (607/1476)	**<0.001** †
BMI (kg/m^2^)	26.0 [23.7–29.3]	25.5 [23.2–29.1]	**0.004** #
**Underlying conditions/comorbidities**			
Hypertension	50.5% (1223/2423)	49.2% (726/1476)	0.435 †
Diabetes	24.5% (593/2423)	29.1% (430/1476)	**0.001** †
Hyperlipidemia	21.2% (489/2308)	18.9% (270/1427)	0.094 †
Smoking	19.4% (459/2364)	13.9% (201/1448)	**<0.001** †
COPD	10.3% (247/2404)	10.0% (144/1438)	0.796 †
Peripheral artery disease	7.8% (189/2423)	7.5% (111/1476)	0.750 †
Pulmonary hypertension	14.5% (351/2423)	21.3% (314/1476)	**<0.001** †
Preoperative CKD	37.4% (906/2423)	38.5% (568/1476)	0.496 †
Preoperative hemodialysis	7.0% (169/2423)	8.6% (127/1476)	0.062 †
Preoperative stroke	19.3% (464/2402)	28.2% (412/1463)	**<0.001** †
Coronary artery disease	27.4% (605/2208)	29.8% (400/1342)	0.123 †
Myocardial infarction	7.4% (178/2398)	6.8% (98/1442)	0.467 †
Septic cerebral embolism	17.7% (430/2423)	25.4% (375/1476)	**<0.001** †
LVEF			
≥50%	70.3% (1639/2332)	78.6% (1122/1428)	**<0.001** †
≥30% to 50%	25.7% (599/2332)	18.7% (267/1428)	**<0.001** †
<30%	4.0% (94/2332)	2.7% (39/1428)	**0.036** †
Alcohol abusus	7.3% (156/2124)	6.4% (83/1294)	0.301 †
Preoperative ventilation	7.5% (182/2423)	11.3% (167/1476)	**<0.001** †
EuroSCORE	11.0 [6.0–17.1]	10.0 [5.0–18.0]	0.062 #
**Microbiology**			
Positive blood culture	61.4% (1487/2423)	67.8% (1000/1476)	**<0.001** †
*Staphylococcus* spp.	36.4% (497/1364)	50.2% (456/908)	**<0.001** †
*Streptococcus* spp.	26.7% (364/1364)	23.8% (216/908)	0.121 †
*Enterococcus* spp.	21.0% (286/1364)	12.1% (110/908)	**<0.001** †
*Other microorganisms*	15.9% (217/1364)	13.9% (126/908)	0.185 †
**Echocardiography**			
Presence of vegetation	57.1% (1384/2423)	66.6% (983/1475)	**<0.001** †
Prosthetic valve endocarditis	33.4% (789/2362)	16.6% (239/1441)	**<0.001** †
Pacemaker associated IE	0.6% (15/2423)	0.7% (11/1476)	0.639 †
**Operative data**			
Ascending aortic/root/arch surgery	24.3% (558/2423)	2.0% (29/1476)	**<0.001** †
Concomitant CABG	12.1% (292/2423)	13.6% (200/1476)	0.172 †
CPB time	104.0 [76.0–152.0]	112.0 [85.0–144.0]	**0.014** #
Crossclamp time	71.0 [51.0–103.0]	71.0 [54.0–95.0]	0.469 #
Redo operation	35.0% (848/2423)	21.2% (313/1476)	**<0.001** †

BMI, body mass index; CABG, coronary artery bypass grafting; CKD, chronic kidney disease; COPD, chronic obstructive pulmonary disease; CPB, cardiopulmonary bypass; IE, infective endocarditis; LVEF, left ventricular ejection fraction. † Chi-square test, # Mann−Whitney-U test. Bold indicates a *p*-value < 0.05.

**Table 2:** Postoperative outcomes of patients undergoing surgery for left-sided IE.

	Aortic Valve IE (*n* = 2423)	Mitral Valve IE (*n* = 1476)	*p* Value
30 day mortality (day 0–30)	14.6% (314/2153)	16.7% (206/1231)	0.095 †
1 year mortality (day 0–365)	29.0% (549/1893)	35.3% (384/1088)	**<0.001** †
Re-exploration	21.7% (527/2423)	20.7% (306/1476)	0.452 †
Postoperative stroke	14.4% (309/2143)	17.7% (233/1319)	**0.011** †
Postoperative hemodialysis	13.9% (320/2299)	19.3% (273/1414)	**<0.001** †
Tracheostomy	7.0% (169/2423)	10.2% (150/1476)	**<0.001** †
ICU stay (days)	3.0 [1.0–7.0]	3.0 [1.0–5.0]	**<0.001** #
Hospital stay (days)	14.0 [9.0–21.0]	13.0 [8.0–21.0]	0.362 #

ICU, intensive care unit; IE, infective endocarditis. † Chi-square test, # Mann−Whitney-U test. Bold indicates a *p*-value < 0.05.

**Table 3:** Independent preoperative predictors for 30 day (logistic regression) and 1 year mortality (Cox regression) in patients undergoing surgery for left-sided IE.

	30 Day Mortality			1 Year Mortality		
	OR	95% CI	*p* Value	HR	95% CI	*p* Value
Age > 65 years	1.554	1.121–2.154	**0.008**	2.046	1.564–2.676	**<0.001**
Male sex	0.718	0.523–0.985	**0.040**	0.718	0.558–0.923	**0.010**
LVEF < 30%	2.537	1.310–4.916	**0.006**			
Coronary artery disease				1.548	1.219–1.967	**<0.001**
Diabetes	1.524	1.109–2.093	**0.009**	1.354	1.063–1.724	**0.014**
Pulmonary hypertension				1.424	1.078–1.882	**0.013**
Alcohol				1.830	1.259–2.660	**0.002**
Preoperative CKD	1.975	1.448–2.695	**<0.001**	1.708	1.320–2.211	**<0.001**
Preoperative hemodialysis				2.099	1.460–3.016	**<0.001**
Preoperative stroke				1.414	1.109–1.803	**0.005**
Preoperative ventilation	2.104	1.398–3.169	**<0.001**	1.537	1.095–2.156	**0.013**
Urological focus				1.410	1.024–1.941	**0.035**
Wound focus				1.461	1.034–2.065	**0.032**
Septic embolism spleen				1.360	1.071–1.728	**0.012**
*Staphylococcus* spp. IE	1.734	1.273–2.362	**<0.001**			
Vegetation	1.817	1.071–3.082	**0.027**			
Prosthetic valve IE	1.536	1.115–2.117	**0.009**			
Redo				1.569	1.236–1.991	**<0.001**
Mitral valve IE				1.329	1.043–1.693	**0.021**

CI, confidence interval; CKD, chronic kidney disease; HR, hazard ratio; IE, infective endocarditis; LVEF, left ventricular ejection fraction; OR, odds ratio. Bold indicates a *p*-value < 0.05.

**Table 4:** Independent preoperative predictors of 30 day mortality in patients undergoing surgery for aortic or mitral valve IE (logistic regression).

	Aortic Valve IE			Mitral Valve IE		
	OR	95% CI	*p* Value	OR	95% CI	*p* Value
Male sex	0.642	0.416–0.991	**0.045**			
LVEF < 30%	4.452	2.182–9.084	**<0.001**			
Myocardial infarction				2.134	1.031–4.415	**0.041**
Diabetes mellitus	1.711	1.136–2.575	**0.010**			
Smoking				0.436	0.220–0.864	**0.017**
Preoperative CKD	1.901	1.283–2.818	**0.001**	3.592	2.287–5.641	**<0.001**
Preoperative ventilation				2.883	1.696–4.901	**<0.001**
*Staphylococcus* spp. IE	1.810	1.225–2.673	**0.004**			
Prosthetic valve IE	1.782	1.206–2.631	**0.004**			

CI, confidence interval; CKD, chronic kidney disease; IE, infective endocarditis; LVEF, left ventricular ejection fraction; OR, odds ratio. Bold indicates a *p*-value < 0.05.

**Table 5:** Independent preoperative predictors of 1 year mortality in patients undergoing surgery for aortic or mitral valve IE (Cox regression).

	Aortic Valve IE			Mitral Valve IE		
	HR	95% CI	*p* Value	HR	95% CI	*p* Value
Age >65 years	1.824	1.259–2.643	**0.001**	2.302	1.557–3.404	**<0.001**
Male sex	0.568	0.401–0.804	**0.001**			
LVEF >50%				0.670	0.450–0.998	**0.049**
Hypertension	0.629	0.435–0.912	**0.014**	1.947	1.251–3.030	**0.003**
Coronary artery disease	1.610	1.165–2.224	**0.004**			
Diabetes mellitus				1.951	1.344–2.832	**<0.001**
COPD	1.614	1.098–2.372	**0.015**			
Preoperative CKD	1.645	1.157–2.339	**0.006**	1.528	1.026–2.274	**0.037**
Preoperative hemodialysis	2.028	1.233–3.337	**0.005**	2.000	1.148–3.482	**0.014**
Peripheral vascular disease	1.964	1.330–2.900	**<0.001**			
PAH	1.495	1.021–2.189	**0.039**			
Alcohol abuse	1.693	1.056–2.716	**0.029**			
Preoperative ventilation	1.864	1.172–2.965	**0.009**			
Urological focus	2.000	1.324–3.020	**<0.001**			
Septic embolism kidney	1.712	1.139–2.574	**0.010**			
Septic embolism spleen				1.468	1.029–2.093	**0.034**
*Staphylococcus* spp. IE				1.735	1.198–2.513	**0.004**
Redo	1.699	1.230–2.347	**0.001**			

CI, confidence interval; CKD, chronic kidney disease; COPD, chronic obstructive pulmonary disease; HR, hazard ratio; IE, infective endocarditis; LVEF, left ventricular ejection fraction; PAH, pulmonary arterial hypertension. Bold indicates a *p*-value < 0.05.

## Data Availability

All data have been incorporated into the article. Additional data will be made available upon request in adherence to transparency conventions in medical research and through requests from the corresponding author.
